# How to eradicate polio in Pakistan: Insights from community health workers

**DOI:** 10.1371/journal.pgph.0002289

**Published:** 2023-08-29

**Authors:** Marium A. Sultan, Svea Closser, Arman Majidulla, Saeed Ahmed, Farah Naz, Sadaf Nayyab, Ayesha Zaman, Muhammad Shafique, Ali Sohail

**Affiliations:** 1 Social and Behavioral Interventions Program, Department of International Health, Johns Hopkins Bloomberg School of Public Health, Baltimore, Maryland, United States of America; 2 Sohail Inc, Peshawar, Pakistan; 3 Zaman Welfare Foundation, Lahore, Pakistan; Johns Hopkins Center for Health Security: Johns Hopkins University Center for Health Security, UNITED STATES

## Abstract

Pakistan, along with Afghanistan, is one of two countries where wild poliovirus is still endemic. Frontline workers (FLWs) are the staff most intimately familiar with both implementation challenges and community context. Harnessing their expertise may be a way to improve the community-polio program interface, which has been a persistent and shifting challenge in polio-endemic areas of both countries. From 2020–2022, we engaged frontline workers in 18 Super High-Risk Union Councils (SHRUCs) in Pakistan through a Human-Centered Design ideas competition. In that competition, teams of polio FLWs identified the most significant barriers they faced in conducting their work, and suggested solutions to those problems—a window into the issues the program faces by the people who know it best. The suggestions of FLWs on how to eradicate polio fell into four main categories. First, there were suggestions to tackle community fatigue by reducing touchpoints, particularly visits solely for data collection. Second, there were calls to improve Primary Health Care in SHRUCs, as a way of addressing community frustrations over an intense focus on just one disease in the context of numerous acute needs. Third, there were suggested ways to increase community engagement through locally relevant channels. Finally, many workers suggested improvements to Human Resources processes and workplace dynamics. Across these ideas, one repeated concept is the need for balance between the intensity of polio activities required for eradication and the provision of other government services, including health services. FLWs engaged the process deeply, providing well thought out problem statements and ideas for change. It is our view that there is no one more qualified to speak to the issues on the ground than FLWs. There are critical insights available if we listen to people who are instrumental to the success of health programs, but not commonly involved with creating policy.

## Introduction

Pakistan, along with Afghanistan, is one of the two countries where wild poliovirus is still endemic. The Polio Eradication Independent Monitoring Board (IMB), in its 17^th^ report, urged development of a “supportive, empowering, problem-solving performance culture for the front line” and encouraged solicitation of feedback from frontline staff on practical difficulties and morale. Additionally, the IMB recommended that “improving communication–in particular, starting with sensitive listening–should be at the heart of the polio programme at every level” [1].

Community-level activities for vaccination programs are usually determined by regional or national level planners. However, frontline workers (FLWs) are the staff most intimately familiar with both vaccine coverage challenges and the community context, as they conduct the day-to-day labor of implementation [2]. Harnessing their expertise could be integral in improving the community-polio program interface, which has been a complex and shifting issue for the polio program in Pakistan and Afghanistan [3, 4].

From 2020–2022, we engaged FLWs in 18 Super High-Risk Union Councils (SHRUCs) in one district in Pakistan though a Human-Centered Design (HCD) ideas competition, asking them to suggest programmatic improvements to the polio program. In these SHRUCs, selected because they were polio endemic or at high risk for polio importation, the Global Polio Eradication Initiative (GPEI) was aiming a ‘laser-focus’ on eradication activities, adopting a vertical approach with paid full-time staff contracted through GPEI partners. In this article, we outline FLWs suggestions and insights—a window into the issues the program faces by the people who know it best.

## Methods

The core of our Human-Centered Design (HCD) ideas competition centered around brainstorming sessions where teams of polio frontline workers (FLWs) identified the most significant barriers they faced in conducting their work, and suggested solutions to those problems. The submitted ideas later went through a shortlisting and selection process by a panel of polio eradication program leaders, and the winning ideas were supported for implementation by leadership.

Each FLW idea brainstorming team consisted of one Area Supervisor (AS), three to four Community Health Workers (CHWs) and three to four Social Mobilizers (SMs)- the same group of women who worked together in the field. Some teams also included Area Level Social Mobilizers (ALSMs). Nearly all FLWs in our study site were women, while the majority of Union Council (UC)-level managers and higher-level officials were men.

We asked participants to describe, in writing, one key problem that negatively impacted their work; what they felt the underlying reason for the problem was; detail a possible solution; and provide an implementation strategy for that solution. We provided direction and guidance on process, but not on content, allowing the scope of problems and solutions to remain wide open. FLWs themselves determined what they felt were the priority issues facing the program.

Our team ran brainstorming workshops directly in five SHRUCs in round one. In round two we expanded to all 18 SHURCs of the district, conducting workshops in three directly, with the rest being led by UC-level managers we trained on the IMPACT process. In the interest of confidentiality, the managers who led the sessions did not read the FLW idea submissions but collected them and passed them on to our team.

Our team read the full text of the submitted ideas, which were handwritten on pages headed by a question prompt. After reading the full text we summarized the problems and solutions in English, onto a spreadsheet, grouping similar submissions together. For the process of longlisting, we shared these sheets (S1, S2 Appendices) along with a scoring rubric for each idea with a panel of polio leadership; this panel included representatives from district and provincial leadership. Members of our IMPACT team also ranked the ideas, and the average scores from the IMPACT team, the district leadership, and the provincial leadership were weighted equally in the final overall score.

Shortlisted teams participated in a pitch competition where they presented their ideas to a panel of polio leaders from district, provincial, and national levels. The panel selected winning ideas for implementation. We continued to work with FLWs and polio leadership throughout the implementation process. Over the course of two rounds of our Human-Centered Design process, we received 189 idea submissions from 171 teams, and nine were chosen for implementation (Table 1).

**Table 1:** Count of frontline worker ideas, by round of HCD process.

	Round 1	Round 2
Unique Ideas	57	132
Winning Ideas	5	4

In this paper, we supplement the material submitted by FLWs in these brainstorming sessions with material from our interviews and participant observation. For participant observation, members of our team accompanied polio workers, at the frontline and management levels, in their daily activities, including door-to-door vaccination, data collection, social mobilization, and data compilation, during the polio campaign cycle of June 2021.

We conducted three rounds of in-depth interviews over the course of the project. We spoke with 63 polio FLWs, managers, and higher-level officials before round one for a diagnostic study; 25 after round one to gather feedback on our process to make improvements; and 28 respondents, at endline, after round two.

### Data analysis

Our starting point for data analysis for this paper were the sheets containing the summarized English translations of ideas, which we had prepared during the idea competition to share with the judges (S1, S2 Appendices). Using the data from these sheets, we grouped ideas with similar themes and created summary statistics. We scanned the handwritten Urdu text of all the idea submissions and read many ideas in full in order to prepare this paper. Our goal was to fully understand the suggested solutions and the underlying problems, and to able to highlight illustrative quotes from FLWs’ thoughtful write-ups.

We engaged with the idea submissions, and our notes from participant observation, inductively, not using any software or codebook.

Interviews, which took place in Urdu, Pashto, or English, were transcribed, and translated into English. We iteratively and inductively coded these interviews in MaxQDA, using a codebook developed through group discussion, and convened regularly over Zoom to share emerging themes and findings.

### Ethics statement

We sought ethical approval from the Johns Hopkins Bloomberg School of Public Health Institutional Review Board, who approved the study as non-human subjects research [IRB00013666].

Informed oral consent was obtained from all participants, as approved by the IRB. We chose to use oral consent to account for varying levels of literacy among study participants, to allow for virtual/distanced interview modalities, and to avoid a risk of a breach of confidentially. This process was entrusted to our trained research team, all of whom have ethical certification in human-subjects research, without an additional witness.

Initial interview participants were recruited in collaboration with local polio program leadership and follow up interview participants were recruited through relationships built during our HCD process. Participants for observation were recruited in collaboration with local polio program leadership. The HCD process was implemented as operational research in collaboration with the polio program (S1 Checklist), and all FLWs from the target UCs were invited to participate.

Notes on participant observation only focused on the participant’s workday and, while not anonymized, did not include any personal information. These notes were not shared outside of the research team. For idea submissions and interview transcripts, identifying information, such as names or contact details, were stored separately using a coded identifier for each FLW team or interview participant.

## Results

Most of the solutions proposed by FLWs to address the challenges of polio eradication fell into four broad themes: tackling community fatigue, strengthening Primary Health Care, community engagement, and issues in the workplace (Table 2). We describe each of these topics in turn below.

**Table 2:** Count of ideas for program improvement submitted by FLW teams.

Section	Idea Submission Topic	Number of Teams
1	**Tackling Community Fatigue**	**35**
1a	Fewer Visits, Fewer Campaigns, and Better Timings for Home Visits	21
1b, 1c	Less Data Collection and Better Data Management	6
	Other	8
2	**Strengthening Primary Healthcare to Achieve Eradication**	**39**
2a	Health Camps	6
2b	Improving Government Clinics	23
2c	Water and Sanitation	1
2d	Campaign Co-Delivery	9
3	**Community Engagement**	**38**
3a	Engaging Religious Influencers	7
3b	Managing Refusals by Doctors and Government Workers	5
3c	Introducing Educational Videos	7
3d	Creating a Polio Dial-Tone	3
3e	Curriculum Changes	1
	Other	15
**4**	**Workplace Issues**	**77**
4a	Reducing Overtime Work	10
4b	Better Processes for Receiving Work Plans	5
4c	More Selective Hiring	10
4d	Enhanced Skills Training	5
4e	Violence from the Community	5
4f	Space to Discuss Workplace Issues	4
4g	Longer Term Contracts	7
	Other	31

### 1. Tackling community fatigue

In our study city in 2021, there were four country-wide Oral Polio Vaccine (OPV) campaigns, four sub-national OPV campaigns, and one case-responsive OPV campaign. Additionally, there was a joint measles and OPV campaign. Thus, each child under 5 years of age in our study area was targeted for OPV vaccination between eight and ten times in 2021.

Yet contact between the polio program and families included more than just vaccination. During a single campaign cycle there could be over half a dozen touchpoints between workers of the polio program and each household (Table 3). There was no standard number of visits, and there were variations in both service provision activities and data collection processes between campaign cycles and coverage locations; Table 3 shows the range of contact between the program and families that could occur.

**Table 3:** Activities and data collection during a polio campaign cycle.

Cadre	Activities during household visits	Data collected	Additional data in the case of refusals
Pre-Campaign			
Community Health Worker/ Social Mobilizer pairs	Data validation, routine immunization check and vaccination referrals; mobilization [creating agreement to vaccinate] for upcoming campaigns; health camps; ’mother’s sessions’, and COVID-19 vaccination	Number of newborns, newly married couples, and transitory residents	Name of head of household, contact number, caste, religion, and tribe
Area Supervisor	Monitoring and verification of information given to, and data collected from, a sampling of households	If the house was visited; what data was collected; and how the by CHW/H2H pair behaved with the family	Reasons for vaccine refusal
Area Level Social Mobilizer	Mobilizing refusals as needed		None, only mobilization
Union Council Level Managers	Validating data provided by Area Supervisors, and mobilizing refusals as needed		Political affiliation
During Campaign			
Community Health Worker/ Social Mobilizer pairs	Vaccination, maintain records, finger-marking, wall-chalking	Number of newborns, newly married couples, transitory residents, guest children. When the child will come back home, in case of missing children	None, only mobilization
Area Supervisor	Monitoring and verification of data, door chalking, and finger marking; referral of pregnant and lactating women to medical center; giving logistical help to the CHW/H2H team	If the house was visited, if requisite questions were asked, how the CHW/SM pair behaved with the family	
Area Level Social Mobilizer	Mobilizing refusals as needed; reporting ongoing refusals to managers		
Union Council Level Managers	Mobilizing refusals as needed; monitoring other cadres; addressing community conflicts		
Post Campaign			
Community Health Worker/ Social Mobilizer pairs	Data validation; Routine Immunization outreach activities	Same as pre-campaign	None, only mobilization
Area Supervisor	Monitoring and staff support		
Area Level Social Mobilizer	Mobilizing refusals as needed		
Union Council Level Managers	Mobilizing refusals as needed, monitoring and staff support		
Between Campaigns			
Community Health Worker/ Social Mobilizer pairs	Mobilization for upcoming campaigns; mobilization for COVID-19 vaccination	Updating micro census book (MCB)	Data collected when requested by district office
Area Supervisor	Monitoring of staff; mobilizing refusals		
Area Level Social Mobilizer	Mobilizing refusals		
Union Council Level Managers	No household visits		

In a single polio campaign, every household was visited multiple times. Many of these visits involved data collection but no service provision. The cycle was repeated for each campaign—eight to ten times in 2021.

Many FLW teams reported that some households experienced these repeated visits as unnecessarily invasive of privacy and personal space. They wrote that repeated visits increasingly caused refusals, as people were tired of responding to so many disruptions to their daily life.

An FLW explained in an interview: *‘The people in our area are getting annoyed with our frequent visits… We get sad but we know it’s our job and we must keep our heads high and give our best [effort]*.*’* FLWs proposed a number of changes to address these issues.

#### 1a. Fewer visits, fewer campaigns, and better timings for home visits

Many FLW teams argued that families would be more likely to accept the vaccine if they were not faced with such frequent visits. Some teams suggested less door knocks during each campaign cycle, with necessary activities being grouped together into single rather than multiple visits.

FLWs had several suggested strategies for reducing knocks. One was that Social Mobilizers, who visited families but did not carry vaccine, should be allowed to administer OPV, removing the need for an additional visit by a CHW once they had convinced a refusal family to vaccinate.

Another suggestion for reducing door knocks was to conduct fewer campaigns per year. One idea submitted was that, when environmental samples were negative for poliovirus, campaigns could be spaced more widely. One team wrote: *‘We need to a have month break between campaigns*. *If there is a campaign in January*, *there should absolutely not be a campaign in February*. *There can be one in the end of March*. *[If we have this gap] parents will not say we are coming too many times for polio and will happily vaccinate*.*’*

Some teams also suggested more careful planning about the best times to approach households. An FLW explained in an interview: *‘I know about the people in my area*, *they wake up late*, *so when we visit their house early in the morning*, *they are uncomfortable*. *Most of the men are still at home and*, *if they open the door*, *they are frowning and sometimes not fully clothed*. *That makes us the villains of the area and can disturb our relationship with the community*.*’*

One specific suggestion made by a team of FLWs was to start the workday at ten in the morning instead of eight, so FLWs do not need to insist children be woken up to vaccinate. Another team suggested not to approach households on Sundays.

#### 1b. Less data collection

Many CHW visits to households were focused specifically on data collection. Large amounts of time and effort were spent to collect, check, and share data by workers at every level of the polio campaign. Each day, during the campaign, workers consolidated and shared data on households they visited, often including demographic details and reasons for refusal (Table 3).

FLWs reported that frequent and repeated questions were seen by some in the community as invasive. They particularly identified this as an issue in households that refused OPV, as those households were targets of additional data collection. FLWs were charged with collecting detailed data from refusal households, including their names, phone numbers, tribe, political affiliations, religious/sect affiliation, and potential influencers (Table 3). The collection of this data could sometimes serve to add fuel to conspiracy theories that the polio program was connected to police or international military operations. FLWs wrote that refusal families feared that this data was being collected for ulterior motives, such as to connect them to militant groups. Thus, FLWs often faced hostility, and occasionally faced violence, during the process of data collection.

One team suggested that a comprehensive survey be conducted to collect all relevant data. That data should be used for future planning and reporting, and no more polio related data collection should be allowed for a certain period to avoid community fatigue from surveys. Other teams suggested stopping the additional detailed data collection from refusal households. They felt this would allow FLWs to focus on core vaccination and communication activities and reduce doubts about the intentions of the polio program.

#### 1c. Better data management

Related to decreasing data collection touchpoints, multiple teams called for better storage and sharing of data, including in digital systems. CHWs and Social Mobilizers collected data in paper notebooks and shared the information they collected with their immediate supervisors at the end of each workday. Supervisors painstakingly input this handwritten data onto digital forms and sent it to area managers.

One team argued that starting a digital data record, which would require both new hardware and additional skills training for FLWs to use, could lessen door knocks. *‘Due to not having a proper data record*, *we must go into the community again and again to get data*, *because of this*, *the community gets irritated with us*, *and they tell us ‘You have taken this data many times before*.*’ If this data was with us on a laptop*, *then the multiple knocks would lessen*.*’* They added that if the full record of each household’s data was digitally available, new workers could easily review previous data, and higher-level managers would always have access to the information.

Another team suggested a mobile application in Urdu for data collection, compilation, and reporting, replacing the intensive documentation and paperwork done manually by FLWs.

### 2. Strengthening primary health care to achieve eradication

When FLWs went door to door, they heard pleas for health services such as medicine, maternal health, and nutrition services. FLWs referred families to government-run centers for these needs, which these FLWs could not provide.

FLWs pointed out that when families went to these centers, they could be disrespected or could receive substandard or no treatment. FLWs said that community members whom they referred to these centers often reported back that they were laughed at or told they were not eligible for services. Frustration that built from not having basic health needs met sometimes took the form of OPV refusals or anger towards polio FLWs.

Most of the neighborhoods in our study site had poor infrastructure and no access to services such as clean drinking water, sanitation, and solid waste disposal. Open sewers ran in front of houses in some neighborhoods, and solid waste was piled up in heaps. These environmental conditions in our study site set up a perfect breeding ground for disease transmission (including polio transmission).

Community members were frustrated by the contrast between frequent OPV campaigns and the lack of health services and systematic underinvestment in sanitation. One FLW team wrote about the complaints and demands they heard: *‘In our area*, *people ask why we don’t do any work apart from polio [vaccination]*, *such as cleaning the neighborhood or providing health services*. *They say “In our area can you install doctors or a free [health] camp*? *When our kids have a cough*, *cold*, *or fever*, *where are you*? *When we go to the government hospital*, *the staff does not behave properly with us*, *they do not provide checkups*, *and they talk aggressively*. *You come here with your head held high*, *while sewer water flows outside*. *Fix the pipes*! *All you do is talk about polio*.*”‘*

Another FLW commented on community frustrations in an interview: *‘There are many needs of the community*, *some ask for food*, *some ask for free medicine*, *some ask for ID cards*. *They don’t have just one need*, *but many*. *We face questions like “Why do you think we need vaccines more than food*?*” and “Why do you concentrate more on vaccination but less on hunger*?*”‘*

Teams tackled various aspects of these issues in their idea submissions, presenting a range of solutions.

#### 2a. Health camps

Multiple teams of FLWs suggested setting up health camps in their area, where medical doctors would provide free consultations and medicines, refer patients to hospitals as needed, and raise awareness about the importance of polio vaccination.

FLWs said it would be helpful to their credibility if they were to inform households about the upcoming camps during their house-to-house visits, and be involved with running the event itself. FLWs felt being associated with these camps would help them be seen as health workers who provided more than just polio drops.

Plans for health camps were already underway when we conducted our HCD process and were implemented over the course of our project. When we conducted participant observation, we heard FLWs advertise these upcoming health camps to the households they visited. They felt proud to be able to do this. In the wrap-up interview after round one, an FLW commented: *‘People receive medicines in the health camps*. *This is great*, *because people thought of us as only good for polio drops and vaccines*, *but now by providing medicines*, *people approach us*, *and listen to our instructions*.*’*

#### 2b. Improving government clinics

Many FLWs highlighted that the poor services at government-run health centers caused the community to mistrust them. A FLW team argued: *‘The community people say that “You people come from the same place [the government health center]*. *When we don’t get the things we need*, *like medicines*, *why should we listen to you*? *Why should we give our children the drops*? *Why should we give them immunization*?*" Then they become OPV refusals*, *and they make things hard for us*.*’*

Another team shared that when community members were mistreated at the government clinics the FLW referred them to, this caused a reversal of their hard work to convince the family to accept OPV vaccination. *“When*, *through a lot of effort we convert a refusal [to accept the vaccine] and refer them to the health center*, *and they are mistreated there and not given a good checkup*, *they again become a harsh refusal*. *The way that the staff treat people causes a lot of [polio vaccine] refusals*.*”* Conversely, FLWs wrote that they felt if community members received respect and care at the health centers, they would treat polio staff with respect and be less likely to refuse vaccination.

Government workers in Pakistan are difficult to terminate without serious cause, which, some FLWs felt, led to them being unconcerned with their job performance. One team suggested incorporating a feedback system for government health centers similar to those existing in private institutions, where complaints are formally responded to by the health department. One feedback method suggested, by another team, was a mobile application where FLWs and community members both could register complaints about negative experiences at government health centers.

Other teams suggested there should be better interpersonal relations training for the government health staff, including skills such as communications and customer service, and that there should be monitoring to ensure that the resources that are meant for community members go to those who are eligible.

Government health centers are of course outside the purview of the internationally run, vertical polio program. Yet improved quality at the government run facilities would likely lead to more trust of polio FLWs.

#### 2c. Water and sanitation

In an interview, a FLW described her frustrations around advising the community on hygiene when there was no clean water available, and how she wished she could help fix this issue: *‘The area I work in doesn’t have a proper sewerage system*. *I would like to fix that issue for the community and provide them with clean water because when I suggest that they should use clean water and stay away from polluted water*, *they respond that they don’t have any source of fully cleaned water*.*’*

One FLW team suggested that the polio program should help coordinate between water and sanitization services and households in the community, helping get their needs met. They felt that if the community was able to present their demands to service providers with facilitation from polio staff, it would increase their trust in the polio program.

#### 2d. Campaign co-delivery

Many teams suggested that FLWs provide medicines, nutritional supplements, maternal care, and other co-delivered services during polio campaigns themselves. One team wrote: *‘*…*we should give out medicines too*. *The community will be happy*, *refusals will be less*, *and more people will say we don’t only work for [the eradication of] polio*, *we care about other needs as well*.*’*

FLWs felt that even small non-health related incentives would make a difference in community perceptions. For example, an FLW team suggested giving educational workbooks to children in line with their age: *‘[If we give out workbooks] community members will believe we care about them*, *and their child’s education*. *If one balloon can make so much of a difference*, *think about how much difference [educational workbooks] can make*.*’*

Incentive programs, providing items such as balloons, soap, or nutritional supplements, had existed on short term bases in the past. When short-term incentive programs started and then stopped, FLWs reported that this could actually increase refusals, as community expectations were raised and then disappointed.

Due to the issues caused when co-delivery programs started and stopped, or when they covered some areas and not others, a couple of teams even suggested not having incentive programs at all. However, many other teams agreed that *consistently* providing services other than OPV would make the community feel more positively about door-to-door visits and more likely to vaccinate their children against polio.

### 3. Community engagement: influencers, media, and school curricula

#### 3a. Engaging religious influencers

The polio program in our study site worked with a team of male religious support persons (RSPs) to engage with families who refused the vaccine and gave religion as a reason. These RSPs give assurances to the community that the polio vaccine is Islamically permissible, and the polio program is not operating against Islamic doctrines.

One FLW team argued for the need for female RSPs, presenting details of the religious pushback that vaccinators face going door to door. They wrote: *‘During fieldwork*, *when visiting the homes of religion-based refusals*, *religious women give us lectures on Islam… They overlook us and don’t vaccinate… Our program should enlist local women religious scholars to spread the word that*, *Islamically*, *the polio program is not wrong*, *that way even people who are religious refusals will vaccinate*. *They should be trained on what polio is*, *and how people get it*, *and the benefits of the drops so they can explain to people the difference between halal (permissible) and haram (impermissible)*.*’* This idea was selected for implementation and taken to the provincial religious scholars’ task force for further discussion.

In addition, six teams submitted ideas related to better hiring and engagement of male RSPs, who FLWs reported had variable levels of commitment to the polio program. Teams suggested increasing incentives, adding intensive onboarding with education about the benefits of OPV, and more selectively recruiting these religious leaders.

#### 3b. Managing refusals by doctors and government workers

Some teams of frontline workers felt that refusals from doctors and government employees were more likely to influence other families to refuse the vaccine. FLWs said they struggled to address these refusals due to the perceived higher status, and therefore social power, of these men and women. Community members from these groups sometimes threatened FLWs with social consequences if they were to document their household as having refused OPV, forcing FLWs into a difficult bind.

One solution selected for implementation was that refusals from doctors and government employees should be handled at a higher level by district or provincial officials of the polio program, working with government officials. The thinking was that officials at these levels would have the clout to directly address government employees refusing the vaccine.

#### 3c. Introducing educational videos

Multiple teams suggested strategies for utilizing media, particularly videos, to spread awareness and educate the public about polio. One team of FLWs suggested documentary-style segments about polio on television. Another team suggested videos in local languages dispelling the idea that there are religiously impermissible or harmful ingredients in the vaccine drops. A third team wrote that when there is fake news about polio online, it should be removed, and accurate information be posted.

#### 3d. Creating a polio dial-tone

In Pakistan during the Covid-19 pandemic, when you made an outgoing call and were waiting for the person to pick up, instead of a ringing noise, you heard a government public service announcement (PSA). These progressed from telling people to wear masks and stay home, to how to register for a vaccine, to informing people to get a booster shot if they got their second dose more than 6 months ago. Pakistan’s Covid-19 vaccination push has ended up being effective, with more than 80% of eligible people vaccinated by April 2022, in part thought to be due to this highly accessible source of information on how to vaccinate [5].

FLWs suggested having an educational dial-tone of this kind to inform people about polio, before and during the campaign days, and this idea was chosen for implementation in round two of our competition. The winning team which submitted this idea pointed out that every household is likely to have access to a mobile phone, which makes dial-tones the form of mass media with the highest potential coverage.

#### 3e. Curriculum changes

Polio cases in Pakistan are currently very rare, with most people never having seen someone affected by it. Some FLWs felt there was not enough knowledge among younger people about polio and suggested adding a segment to school curriculums to cover this gap: *‘There should be a mention of polio in the curriculum…This generation should know what polio is and what harms it has*.*’*

### 4. Workplace issues: reducing overtime work, improving hiring and training, and tackling violence from the community

#### 4a. Reducing overtime work

One winning idea in round one was reducing overtime work. FLWs wrote that they had to respond to phone calls and text messages outside working hours, primarily for the purpose of sharing data.

When tracking FLW activities during the campaign month immediately following implementation of this idea, we saw that after-hours work had almost entirely halted. However, talking to FLWs months later, some, but not all, of them said that requests for data outside working hours had returned, signaling that changes to campaign operations need ongoing commitment to maintain. *‘Our working hours are fine*,*’* one respondent commented, *‘but we receive calls late at night about the data we have collected*.*’* Another respondent clarified, *‘Not* ***always***, *but sometimes*.*’*

#### 4b. Better processes for receiving work plans

Multiple teams of FLWs suggested they receive work plans and other similar instructions in a uniform fashion, as at times they received conflicting instructions from various sources, for example supervisors and external monitors. FLWs proposed documenting all operational instructions in an up-to-date manual they would be able to refer to. This idea was selected for implementation in round two of our competition.

#### 4c. More selective hiring

Some teams of FLWs suggested that hiring processes could be improved. They argued that FLWs hired with appropriate education and experience were best for high quality campaigns, and for positive community relations. They suggested adopting more stringent hiring criteria and ensuring that workers are hired on qualifications and not favoritism.

Other teams added that even if full-time FLWs were qualified, the “volunteers” that were hired on a temporary basis to fill in staffing gaps were sometimes could not do the work to the high standards they would have liked to see. Teams wrote that properly trained and supported CHWs would reduce the teaching and monitoring burden on their immediate supervisors. They made a sports analogy to underline the value of training extra staff as reserve for when regular workers were not available: *‘On a cricket team there are 11 players playing*, *but 15 are ready—in case one must step in for another*.*’*

#### 4d. Enhanced skills training

Many FLWs, in their idea submissions and during interviews, said that they were unable to answer some of the questions they were asked about the vaccine and the program. FLWs did not always know the answers to questions about vaccine ingredients, the need for a cold chain, and vaccine safety, and felt that at times they were unable to counter viral rumors. FLWs further argued that an inability to answer questions completely and accurately affected their credibility in the community, fueled mistrust, and had a direct impact on the program.

They suggested enhanced training addressing such knowledge gaps in depth, so that FLWs could respond to all questions from the community. Specifically, they suggested a standard narrative for common questions, and an improved training methodology to accommodate the needs of lower educated FLWs. This idea was chosen for implementation in round one.

On top of this knowledge building around communications, other teams of FLWs said they want to learn additional skills including self-defense; professional vocabulary and communication in both English and Urdu; and software such as MS Office.

#### 4e. Violence and harassment from the community

A significant problem FLWs in our study site faced was violence and harassment from community members as they went door to door. This is a complex and pervasive issue. In our interviews, participant observation, and idea submissions we learned that FLWs faced severe, intermittent violence, on top of more regularly occurring harassment and disrespect. Several teams of FLWs related they believed a lack of consequences for perpetrators was a contributing factor. FLW teams called for increased legitimacy and support from the government. They suggested documentation such as ID cards, and public statements, delivered via advertising and news programs, affirming that they are government workers. In addition, they wanted more tangible support when violent incidents occur, such as help in filing police reports and taking cases to court.

One team suggested the mechanism for this support could be a dedicated lawyer assigned to polio workers: *‘In the polio program there should be a lawyer assigned from the [district office]*. *When someone hits us or there is a security issue in the field*, *they should deal with the police and legal system themselves*, *so we don’t make enemies*.*’* It’s important to note that FLW ideas in sections one, two, and three of this paper would also contribute to improving community relations, and likely by extension a decrease in harassment and violence.

#### 4f. Space to discuss workplace issues

FLW teams wrote about wanting spaces to discuss problems at work confidentially and openly. Multiple teams suggested that FLWs should have regular private meetings with external monitors, leadership, and/or HR where workers could discuss workplace issues and come up with solutions. Other teams suggested group discussions should take place where frontline workers share common issues and potential solutions.

One team suggested that a hotline staffed by women should be created where FLWs could report workplace issues, including harassment, without facing repercussions. Although this was not chosen as a winning idea, information about an existing, but little known, hotline was better communicated to FLWs in the SHURCs of our study district where they could openly and confidentially discuss issues they are facing.

#### 4g. Longer term contracts

When we began this project in 2020, FLW contracts expired every 3 months. This generated significant stress for many workers, who said they constantly worried about contract renewal. A team wrote: *‘We want our job to be a permanent government position so we can be calm and do our work feeling safe*. *This [job insecurity] has such a bad effect on us at work and at disrupts our home life*.*’*

FLWs argued that short term contracts hindered planning for their children’s education and house rent as these expenditures were based on an annual commitment: *‘We are saving our country’s future*, *but we are not able to secure our children’s future…If our work ends*, *what will happen to our kids*?*’*

Implementing longer-term contracts was a winning idea in round one. Although many FLWs had asked for permanent or annual contracts, the timelines of existing funding and contracting mechanisms made this impossible. Still, contracts for FLWs in the polio program were increased from 3 to 6 months, a major improvement for many workers.

## Discussion

Human-Centered Design is a promising strategy for more equitably planning health systems [6, 7]. However, is important to be thoughtful about creating an inclusive process across power levels, and building-in stakeholder engagement from the beginning [8]. The Pakistan polio program has broadened, expanded, and institutionalized the process we describe here, and is now collecting insights from FLWs from across the country [9]. This institutionalized process is likely to lead to broader, deeper insights from frontline workers, and the insights are more likely to create real change. The program’s commitment to listening to and responding to the needs of its FLWs is laudable.

Some clear policy recommendations emerge from the ideas of FLWs. In their calls for action in these areas, they are not alone; their observations on reducing campaign fatigue [10], the need for integration [11–17], and increasing community engagement [18–20] are echoed in the literature, as well as in recommendations and programmatic changes underway from within the polio program itself.

Across many of these ideas, one policy recommendation that presents strongly is the need for balance between the intensity of polio activities and the provision of other basic government services, including health services. SHRUCs often lack government services such as clean water, sanitation, or Primary Health Care. If community members were to have positive experiences at government health centers, this would have broad-based impacts on improving trust, including in the polio program.

Here, FLWs join academic observers and the Independent Monitoring Board on calling strongly for the polio program’s concerted involvement in integrated services [1, 10–12, 14, 21, 22]. This could be a range of steps, FLWs suggested: including other services in campaigns (and making sure those services are long-term and not one-off); pop-up health camps; improving sanitation; campaign co-delivery with medicines; provision of basic nutrition for children; and working to support the improvement of government clinics. As frontline workers in Pakistan’s SHRUCs are the actors most intimately familiar with community relations and polio operations in these areas, we believe this call should be taken seriously. Many of these strategies are already being implemented by the polio program. The issues involved are not simple for the polio program to address, but taking steps towards provisions of basic services would serve the GPEI very well towards their goal of eradication.

The deep engagement of FLWs in the process of idea generation is worth commenting on. FLWs engaged the process enthusiastically, providing well thought out problem statements and ideas for change—our brief summaries here do not do justice to the depth of their ideas.

The eradication of polio has proven challenging—in Pakistan especially [23]. But it is our view that there is no one more qualified to speak to the issues on the ground than FLWs. It became very clear to us through the course of this work that the polio program’s frontline workers are committed, intelligent, thoughtful women. That their insights are being brought into the polio program’s policymaking is a pathbreaking step that could be a model for other programs.

## Supporting information

S1 Checklist Inclusivity questionnaire. (DOCX)

S1 Appendix Spreadsheet containing summarized ideas from round 1 of IMPACT. (XLSX)

S2 Appendix Spreadsheet containing summarized ideas from round 2 of IMPACT. (XLSX)
